# Forest management strategy affects saproxylic beetle assemblages: A comparison of even and uneven-aged silviculture using direct and indirect sampling

**DOI:** 10.1371/journal.pone.0194905

**Published:** 2018-04-10

**Authors:** Klara Joelsson, Joakim Hjältén, Heloise Gibb

**Affiliations:** 1 Department of Wildlife, Fish, and Environmental Studies, Swedish University of Agricultural Sciences, Umeå, Sweden; 2 Department of Ecology, Evolution and Environment, La Trobe University, Melbourne, Australia; Università degli Studi di Napoli Federico II, ITALY

## Abstract

Management of forest for wood production has altered ecosystem structures and processes and led to habitat loss and species extinctions, worldwide. Deadwood is a key resource supporting forest biodiversity, and commonly declines following forest management. However, different forest management methods affect dead wood differently. For example, uneven-aged silviculture maintains an age-stratified forest with ongoing dead wood production, while even-aged silviculture breaks forest continuity, leading to long periods without large trees. We asked how deadwood-dependent beetles respond to different silvicultural practices and if their responses depend on deadwood volume, and beetles preference for decay stages of deadwood. We compared beetle assemblages in five boreal forest types with different management strategies: clearcutting and thinning (both representing even-aged silviculture), selective felling (representing uneven-aged silviculture), reference and old growth forest (both uneven-aged controls without a recent history [~50 years] of management, but the latter with high conservation values). We collected beetles using window traps and by sieving the bark from experimental logs (bolts). Beetle assemblages on clear-cuts differed from all other stand types, regardless of trapping method or decay stage preference. Thinning differed from reference stands, indicating incomplete recovery after clear-cutting, while selective felling differed only from clear-cuts. In contrast to our predictions, early and late successional species responded similarly to different silvicultural practices. However, there were indications of marginal assemblage differences both between thinned stands and selective felling and between thinned and old growth stands (p = 0.10). The stand volume of early decay stage wood influenced assemblage composition of early, but not late successional species. Uneven-aged silviculture maintained species assemblages similar to those of the reference and old growth stands and might therefore be a better management option when considering biodiversity conservation.

## Introduction

A vast majority of ecosystems across the globe are today shaped by anthropogenic activities rather than natural disturbances [1]. Over the last century, human use has extensively modified most of the world's forests, often accompanied by the deterioration of environmental conditions [2, 3]. These changes in ecosystem structures and processes have led to habitat loss and species extinctions [4, 5].

Through simplification and homogenization of forest structure, even-aged silviculture (e.g., clear-cutting and subsequent thinning) has been linked to severe negative consequences for forest biodiversity [5, 6] particularly for deadwood-dependent species [7, 8]. A conventional managed forest supports only 10% of the deadwood volume occurring in an unharvest forest. Since the large-scale introduction of even-aged forest management in Scandinavia in the mid-20^th^ century, the deadwood volume in managed forests has decreased to an average of 5–7 m^3^/ha, compared with 70–100 m^3^/ha in natural forests [9, 10]. Clear-cutting, with approximately 90–95% tree-removal, creates homogenous forests that lack mature trees for long periods and are harvested before trees begin to senesce, resulting in limited deadwood production [11, 12]. In contrast, a natural boreal forested landscape would support a diversity of habitats with different structural dynamics and deadwood created by wildfire, wind, and tree senescence [13]. Although favourable for some species, the loss of forest continuity and decreased habitat diversity and deadwood volumes are associated with decreased biodiversity [14–16]. New management methods that better mimic natural disturbances and processes are needed to mitigate further loss of biodiversity [17, 18].

Alternative silvicultural methods that strive to balance forest production with conservation of biodiversity by maintaining the structures and processes of natural ecosystem have been developed in various parts of the world, from tropical to boreal biomes [19, 20]. One such method is uneven-aged silviculture. Historically, boreal forests have been structured by both large-scale stand replacing disturbances, e.g. fire and storms, as well as small-scale disturbances, e.g. gap dynamics [10, 21–23]. This created a mosaic landscape with both between- and within-stand variability, thus yielding a multitude of ecological niches and living conditions for many different organisms. Uneven-aged silviculture aims to mimic natural small-scale disturbances, e.g., gap dynamics, and may therefore have a limited impact on species associated with old growth forest characteristics. This is because forest continuity, a stable microclimate and a stratified forest is maintained. Recent studies from boreal forests support this assumption [19, 24, 25]. Reduced destruction of deadwood during harvesting and post-harvesting processes and continuous deadwood recruitment due to tree senescence in uneven-aged stands provides a more beneficial deadwood regime compared with even-aged silviculture [26]. Species differ in their preference for sun-exposure and maintenance of shaded conditions might benefit species adapted to mature boreal forest with long continuity [25]. Thus, uneven-aged silviculture might provide a good habitat for species dependent on old-growth conditions and deadwood. However, our knowledge on effect of uneven aged silviculture on deadwood-dependent species in the boreal forest is limited and further studies on this topic should have the highest priority [18]

About one fourth of all forest species and half of the beetles in Fennoscandia are saproxylic, i.e., dependent on deadwood [8, 21]. This dependence can be through direct consumption of the woody substrate, or indirect, through dependence on other saproxylics, for example fungivores, predators or parasitoids feeding on organisms restricted to the wood [27]. Saproxylic beetles often specialize on particular deadwood types and the communities shift with factors such tree age, tree species, sun exposure, log size and decay stage [8]. Since saproxylic species are sensitive to forest management, the group is highly suitable for evaluating silvicultural effects on biodiversity. Silviculture affects both the quality and quantity of deadwood, so species associated with different decay stages of deadwood might differ in their responses to forest management. Decomposition progresses rapidly in the early decay stages, so early decay stage specialists are often fast colonizers [8, 28]. As decay progresses, the chemical composition and nutrient availability change [29] and decay stages last longer, e.g., it can take up to 50 years for a spruce log to reach the advanced stages of decay [30]. This means that rapid recolonization becomes less important for species dependent on late decay stages [31, 32], but that resources may be much rarer following silviculture, increasing the vulnerability of these species. Previous research from our study system indicated that even-aged forest management has limited impacts on beetle assemblages compared with uneven-aged forest management [24, 25]. Here, we test if beetles using deadwood of different decay stages, and thus with different life histories, respond similarly to silvicultural treatments and if stand characteristics influence beetle assemblages. We also test for consistency between sampling methods by using two different methods of sampling saproxylic beetles, one direct and one indirect.

We asked if forest management strategy, i.e., different silvicultural practices and conservation measures, affected the assemblage composition of saproxylic beetles. We compared assemblages of saproxylic beetles in even-aged silviculture, uneven aged silviculture and unmanaged forest with and without documented high conservation values. We predicted that: 1) forest management strategy affects saproxylic beetle assemblages; and that 2) effects are consistent among trapping methods (sieving of bark from experimental logs and window traps), but that 3) effects are greater for beetle species associated with late than early decay stages of deadwood; and that 4) stand characteristics, e.g., deadwood availability, also influence beetle communities.

## Methods

### Ethic statement

No specific permissions were required for these activities. Insects sampling do not require an ethical permit in Sweden. All landowners were informed and in agreement that we could use their land for our study. Two locations are protected by the Swedish county board. We obtained permission to sample there by Länsstyrelsen Jämtland, through Sara Toivanen, permit number 521-3849-2014.

### Experimental sites

The experimental sites were located in the boreal forest of central Sweden (63.0–62.3 N, 15.2–16.4 W). The landscape is dominated by forest that is largely managed; about 77% of the landscape is forest-covered and 3.5% of the forest is legally protected from forestry [33]. All experimental stands were selected for similarity in tree species composition (i.e., greater than 70% of stand volume was Norway spruce (*Picea abies* (L.) Karst)), understory vegetation and soil properties.

**Fig 1:**
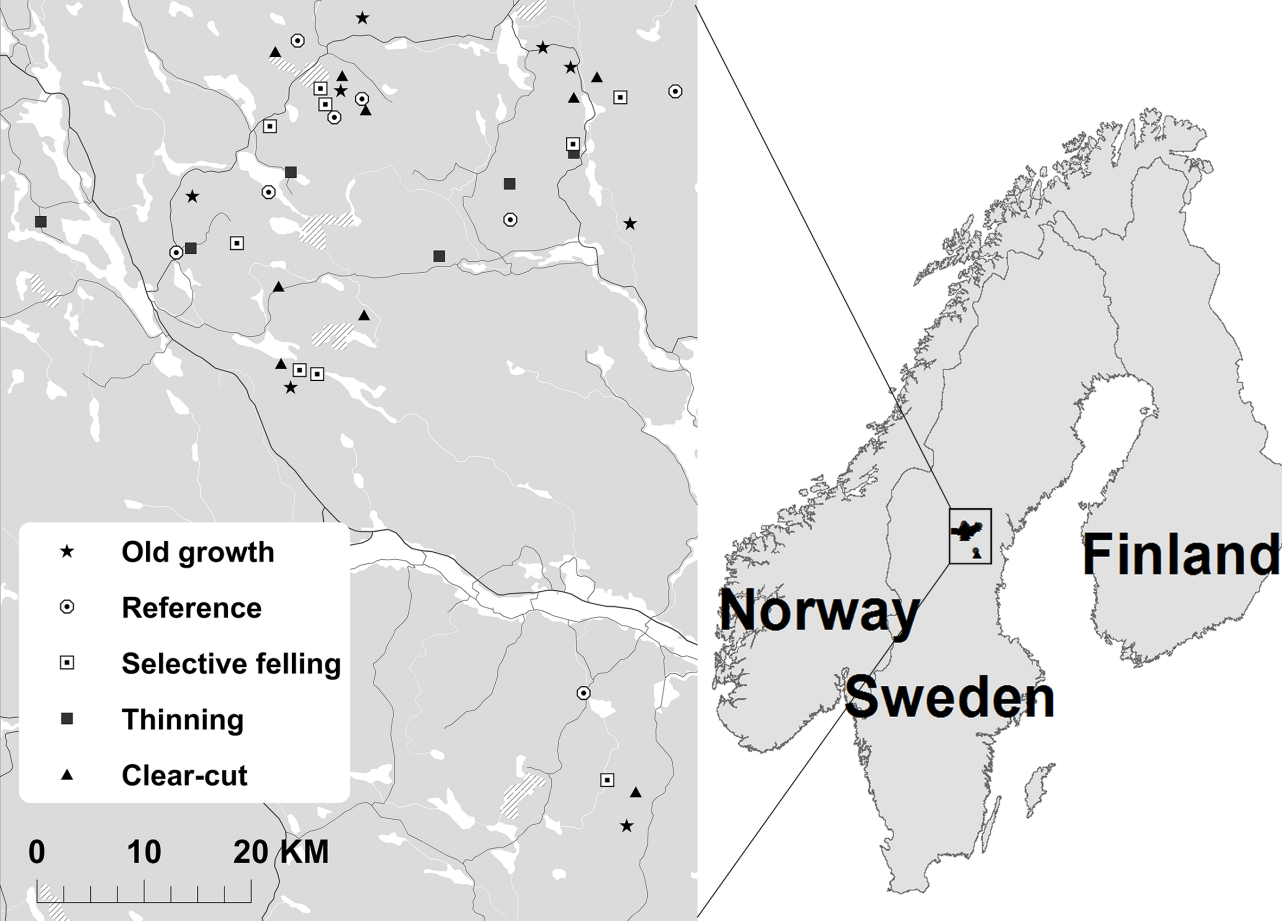
Map over the study area. Grey areas is land, white represents water and the bigger road are marked in black.

**Fig 2:**
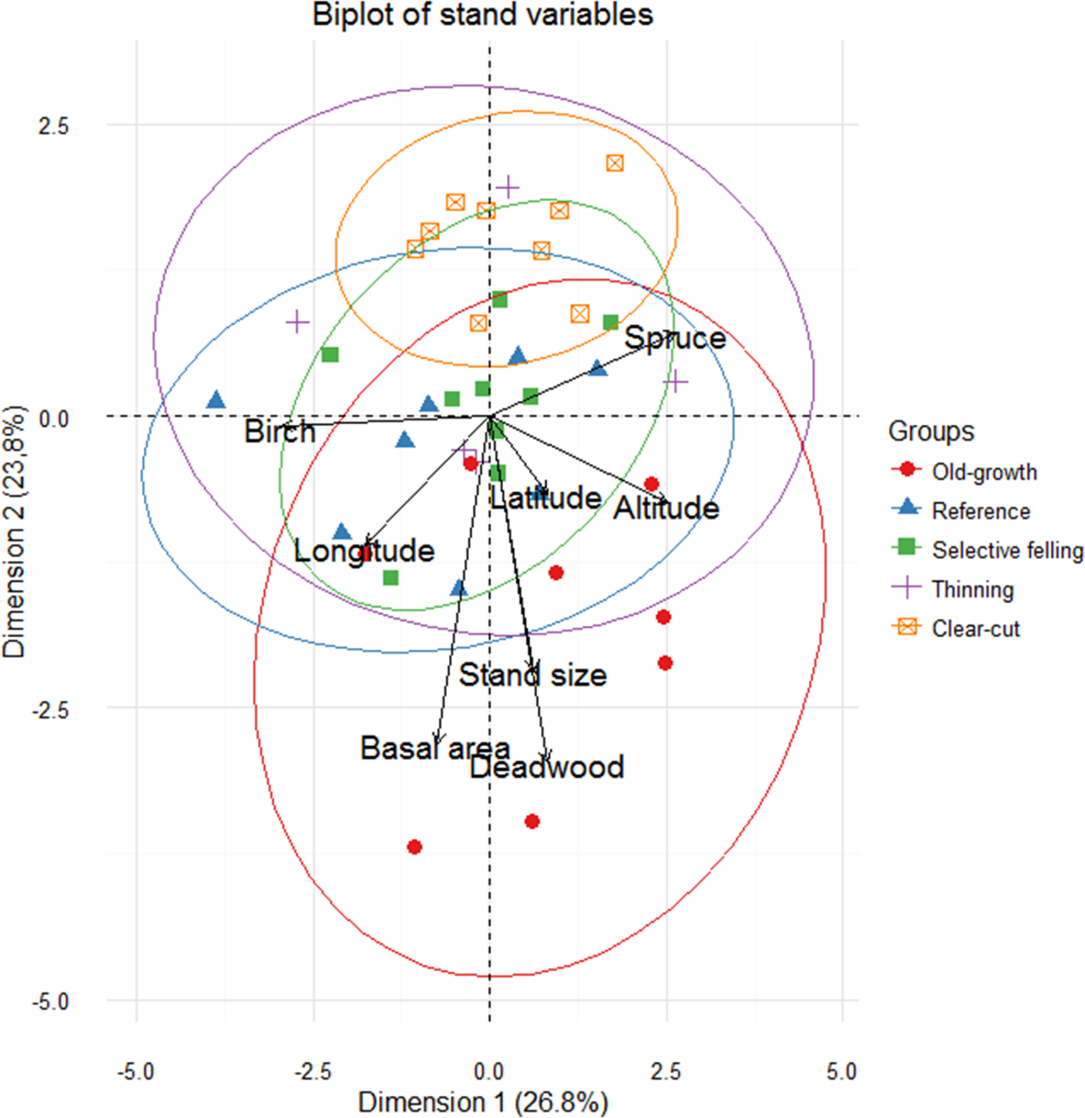
Biplot from principal component analyses (PCA) of the stand variables. The first and second axes of the PCA are shown. The length of the variable arrow corresponds to the degree of explanation. The circles provide 95% CI of treatment centroids. Deadwood volume refers to the total amount.

We compared beetle assemblages in five different forest types: (1) recently clear-cut stands that were uneven-aged prior to harvesting, but became even-aged as a direct consequence of clear-cutting (‘Clear-cut’); (2) older, even-aged stands regenerated after clearcutting 50–60 years ago that have undergone commercial thinning with full timber extraction (‘Thinning’); (3) mature stands originating from uneven-aged, stratified stands which have recently undergone uneven-aged silviculture, where approximately 30% of the standing volume was harvested (‘Selective felling’); (4) mature stands originating from uneven-aged, stratified stands without a recent history (~50 year) of management (‘Reference’); and (5) old-growth forest with confirmed high nature values (‘Old growth). The different stand types were spatially interspersed and no stands were closer than 1000 m apart (Table 1, Fig 1). Managed stands (clear-cutting, thinning and selective felling) varied in the time of harvesting, ranging from 2–14 years prior to our study. An overview of stand properties is presented in Table 1 and further visualized in Fig 2. For a more detailed description of the study system, see [25] and [24].

**Table 1:** Overview of the 39 experimental sites. Mean ± SE given for each of the environmental variables. We conducted pairwise comparisons for the tests showing a significant effect of management strategy. Deadwood volume of spruce (m^3^/ha) was divided into classes based on decay stage and substrate type.

Stand type	Old growth	Reference	Selective-felling	Thinning	Clear-cutting	F-value	DF	p	pairwise
Original state	Uneven	Uneven	Uneven	Even	Uneven				
Current state	Uneven	Uneven	Uneven	Even	Even				
Number of stands	8	8	9	5	9				
Size (ha)	19.5 ± 9.0	7.2 ±1.6	8.0 ± 0.7	7.7 ± 3.0	5.7 ± 1.3	1.7	4	0.2	
Altitude (masl)	418 ± 23	365 ± 25	391 ± 9	366 ± 34	376 ± 18	1.2	4	0.3	
Years since harvest	NA	NA	7.4 ± 1.7	6.2 ± 2.2	6.7 ± 1.8	0.3	2	0.8	
Basal area (m^2^)	25.7 ± 2.9	24.5 ± 1.0	17.9 ± 1.0	18.5 ±2.3	0.3 ± 0.1	43.2	4	**<0.01**	OG,R> SF,T> CC
Spruce % of BA	80.8 ± 6.0	80.3 ± 3.7	80.0 ± 4.1	83.2 ± 7.1	NA	0.0	3	0.9	
Birch % of BA	5.0 ± 2.3	14.2 ± 3.4	13.4 ± 2.1	8.0 ± 3.0	NA	2.4	3	0.09	
Deadwood (m^3^)	46.2 ± 11.4	16.9 ± 4.2	13.0 ± 3.5	4.6 ± 1.0	8.5 ± 3.2	15.2	4	**0.03**	OG> R,SF> T,CC
Early decay logs	10.8 ± 2.8	3.7 ± 0.7	2.8 ± 1.0	2.9 ± 0.6	2.0 ± 0.9	5.8	4	**<0.01**	OG> R,SF,T,CC
Late decay logs	17.4 ± 4.7	3.3 ± 1.1	6.1 ± 2.0	0.7 ± 0.3	4.9 ± 1.6	5.7	4	**<0.01**	OG>R,SF,T,CC
Early decay standing	11.8 ± 2.1	5.1 ± 1.6	2.6 ± 1.0	1.1 ± 0.4	1.0 ± 0.5	10.9	4	**<0.01**	OG>R,SF,T,CC; R>T,CC
Late decay standing	6.2 ± 1.8	4.7 ± 1.3	1.6 ± 0.6	0	0.6 ± 0.3	5.7	4	**<0.01**	OG,R >SF, T,CC

OG = old growth, R = reference, SF = selective-felling, T = thinning, CC = clearcut.

### Sampling

To test whether emerging beetles and species actively flying around in the habitat respond similarly to management strategy, we used two trapping methods, bark sieving and window trapping. Bark sieving has the advantage of collecting species directly associated with a specific dead wood substrate, thus providing invaluable knowledge on substrate preferences. However, the disadvantage with this method is that a limited subset of the species found in a forest stand is sampled. For example, using fresh deadwood, most specimens collected will belong to species associated with early decay stages [34, 35]. In contrast, window traps are the most efficient method for ensuring large samples in terms of species richness and abundance [36–38], but there are concerns regarding the extent to which trapped beetles represent transient visitors that may not necessarily be related to local stand conditions [39, 40]. Thus, a combination of both methods is often recommended to ensure that the species pool captured in flight corresponds to the one emerging from substrates [38, 41]. We therefore used both sampling methods to evaluate if they provide consistent results for beetle responses to forest management strategy.

Experimental logs: In early June 2014, we placed short sections of freshly cut spruce logs from healthy living trees (bolts) in all stands. The bolts originated from a limited number of trees harvested at one location and thereafter transported to the study sites in pieces. In each study site, we haphazardly distributed five bolts, approximately 40 cm long and with a diameter ranging from 20–30 cm. After two summer seasons, at the end of August 2015, the bark from all logs was removed, broken into 5 cm x 5 cm wide pieces and sieved for 5 min. Beetles observed on the wood or bark after bark removal were collected. All visible animals were collected as we sorted the bark in the lab, after which the bark was placed in Tullgren funnels under lightbulbs (840 lm, 53 watt, 2700 K, E27, 230 V) for 24 hrs to drive any remaining beetles out from the bark. We transferred all collected beetles to jars with 70% ethanol solution.

Window traps: Beetles were collected using window traps from June to September 2014, see [25]. We used three traps per stand, placed at 25 m from the stand’s centroid in N, SW and SE directions. The traps were free-hanging triangular window traps with semi-transparent plastic flight intercepts of 0.35 m^2^. The traps hung on ropes strained between two trees or, in some clearcuts, between wooden poles. Beetles were collected in 600-mL plastic bottles one-third filled with 50% propylene glycol and later transferred to 70% ethanol solution.

All collected beetles were identified to species by taxonomic experts, with the exception of the genera *Epuraea*, *Acrotrichis* and *Gabrius*, and classified as saproxylic or non-saproxylic based on ecological classifications [42, 43]. These designations were expanded to include species found in northern parts of Sweden (Hilszczański, J., Pettersson, R. and Lundberg, S. pers. comm.). We restricted our analyses to the obligate saproxylic species, i.e., species that are dependent on deadwood in at least one stage of their life-cycle [8]. Nomenclature and taxonomy of the beetles follows the Swedish taxonomic database (S1 and S2 Tables) from the Swedish Species Information Centre.

### Analyses

We tested the effect of stand management on beetle assemblages using data from both the bolts and the window traps. To test if the effects of forest management on beetle assemblages are consistent among trapping methods, we extracted the 28 species collected in experimental bolts as a subsample from the window trap data. To test if the effect of forest management strategy was greater for beetle species associated with late than early decay stages of deadwood, we divided the total window traps data into two categories, based on early or late decay stage preference. The deadwood preference follows [44] and [45], but since the classification was not originally created for boreal forests, we only used two broad groups: early and late. We defined early decay stage preference as all species with a decay class preference below 3 (dead wood still hard) and late as species with a decay class preference from 3 and above (wood has started to soften). That provided us with four data sets: 1) data from bolts, 2) “bolt species” subsampled from window trap data, 3) early species captured with window traps and 4) late species captured with window traps.

We used manyGLM [46] to test the effect of forest management strategy in the beetle assemblage for the four data sets separately. ManyGLM calculates the parameter estimates of generalised linear models fitted to each of many variables simultaneously. The sum of log-likelihood is calculated for the univariate GLMs to create a test statistic verified through randomization. ManyGLM provides increased statistical power for detecting differences in communities of less abundant species that may be poorly represented by distance-based approaches. Statistical significance was evaluated using 999 resampling iterations via ‘Probability Integral Transform residual bootstrap’ (PIT-trap) resampling fitted using negative binomial link functions [47, 48]. Altitude and stand size were tested as covariates and since they had a significant influence on the model for two of the data sets, they were included in the final model: *beetle assemblage~ management strategy + altitude + stand size*. When management strategy had a significant effect, we performed pairwise comparisons. Univariate tests for all species were conducted for the full models. Since the number of comparisons was large, adjusted comparisons would severely increase the risk of type II errors. Therefore, we used unadjusted P-values for post hoc tests. However, interpret the results with caution [49, 50]. We extracted and compared the species with a significant univariate test for the bolts and comparison from the window traps for additional exploration of potential differences between trapping methods. To visualize the differences in beetle composition, we created Non-Metric Multidimensional Scaling (NMDS) plots based on Bray-Curtis distances on square-root transformed data, with 20 random starts to find a stable final solution [51].

We fitted linear models to test if altitude and stand size varied depending on management strategy. We also fitted linear models to test for management strategy effects on tree species composition, basal area and log-transformed deadwood volume. Since the quality of deadwood might have a higher impact than the quantity on beetles assemblages [31, 52], we divided deadwood into decay stages and substrate types to compare effects of management strategy on deadwood type using the R package ‘nlme’. A principal component analyses (PCA) were performed to assist in the interpretation of effects from management of stand characteristic using the R-package ‘ factoextra’ [53].

To examine how stand characteristics influence beetle communities, we fitted stand variables to the NMDS plots using *envfit* from the R-package ‘vegan’. We used the stand variables presented in Table 1, with exception of years since harvest. The significance test was based on 999 random permutations of the data. Because they were so different structurally, we excluded clear-cuts and repeated the analyses in order to explore which stand characteristics were important in the four forest types with closed canopies. All analyses were conducted in R software version 3.4.0.

## Results

From the bark samples of the bolts, we extracted 6622 individuals belonging to 28 obligate saproxylic species (S1 Table). Of those, 57% were cambivores, 25% fungivores and 14% predators. The corresponding abundance for the same species collected from window traps were 8228 individuals.

**Fig 3:**
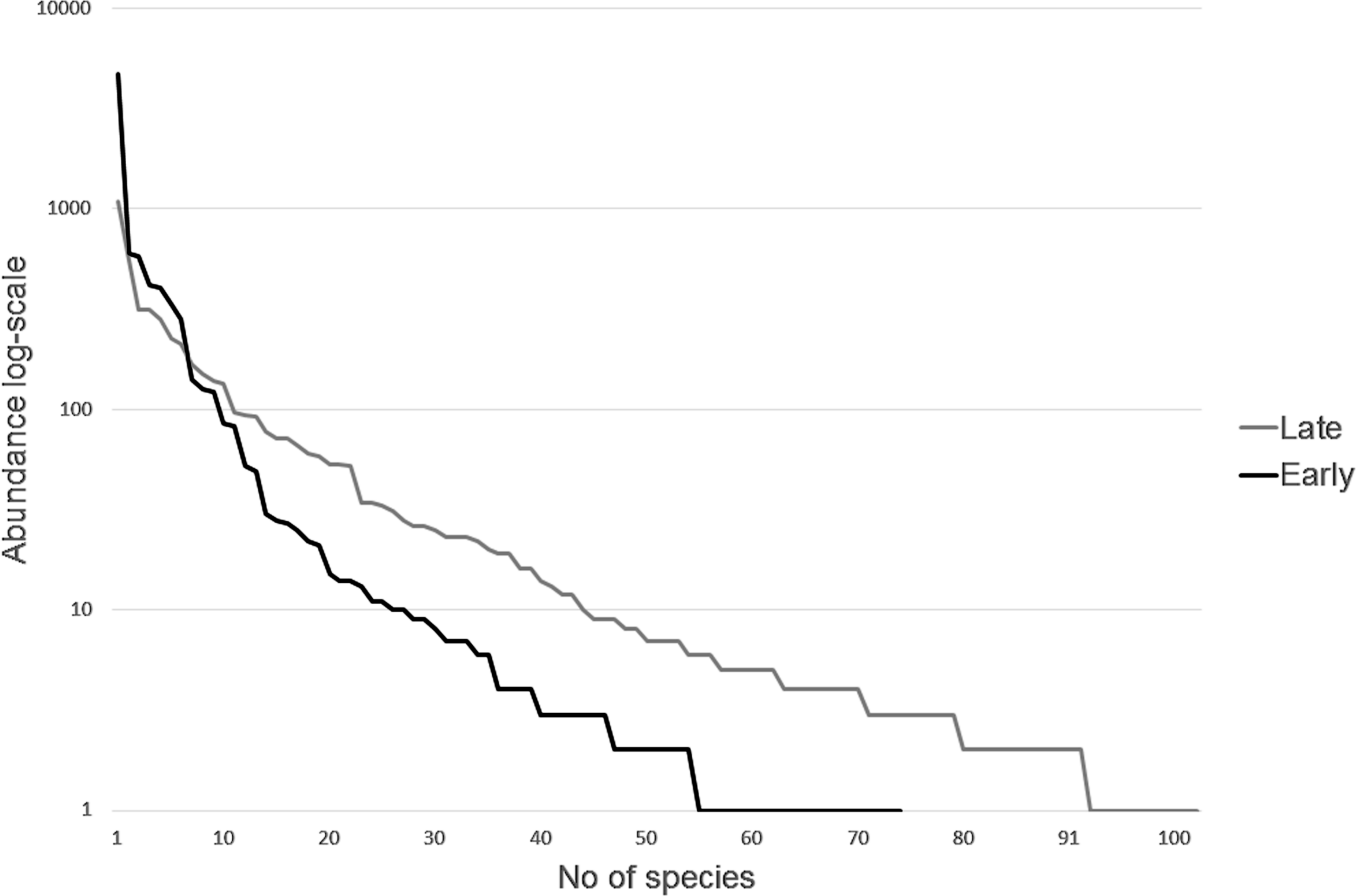
Rank abundance. Rank-abundance comparison between early (decay class preference <3; dead wood still hard) and late successional species (decay class preference ≥3; wood has started to soften) assemblages from window traps.

Window traps captured a total of 14811 individuals belonging to 220 obligate saproxylic species (S2 Table). We were able to classify 80% of species (91% of the total abundance) according to decay class preference; 75 species were associated with early and 103 species with late decay stages. Cambivores dominated the early stages, while fungivores dominated the late successional species, indicating that, as the decay proceeds the species utilizing the deadwood have other feeding strategies. Of the early successional species, 50% t were cambivores 35% predators and 12% fungivores. The remaining 3% of the species were woodborers, detrivores or unknown. Sixty percent of the late successional species was fungivores. In addition, 26% were predators, 12% woodborers and only one cambivore species (*Judolia sexmaculata*) was a late succession specialist. In addition to the shift in feeding guilds, we also saw structural differences between the early and late successional assemblages. The early communities were dominated by a few, highly abundant species while the late succession community had more even abundances of species. The ten most abundant species constituted 91% and 67% of the total abundance for the early and late successional species, respectively (Fig 3).

In total, we captured 8 red-listed species: *Platysoma lineare* (NT), *Eblisia minor (NT)* and *Denticollis borealis (NT)* on clearcuts; *Mycetophagus fulvicollis* (NT) in both thinning and clearcut; *Pytho abieticola (VU)* in selective felling; *Orchesia fasciata (NT)* in selective felling and old *growth; Enicmus planipennis (NT)* in old growth; and *Rhizophagus grandis* (NT) in reference stands. All red-listed species were collected at low abundances, ranging from singletons to a maximum of five individuals.

### Forest management strategy effects on saproxylic assemblages colonizing fresh dead wood

**Fig 4:**
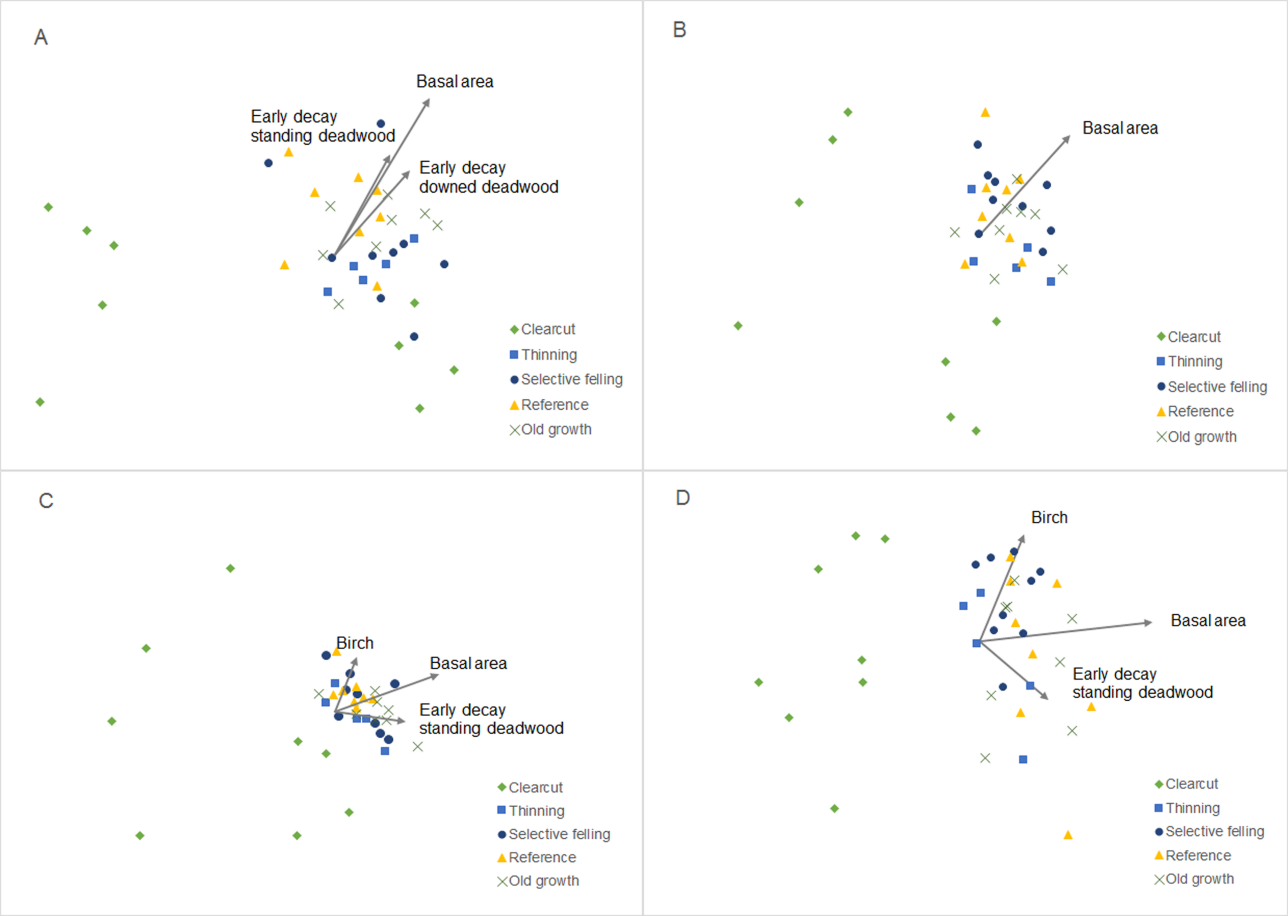
NMDS plots. NMDS plots showing the effect of stand type on beetle assemblages for the four different data sets: A) bolts (stress = 0.15), B) window traps (stress = 0.11), C) early early successional species (stress = 0.12) (decay class preference below 3; dead wood still hard) and D) late successional species (decay class preference 3 and above; wood has started to soften) (stress = 0.15). Data were square root transformed with Bray-Curtis distances and were run with 20 random starts to find a stable final solution. The fitted stand variables with a significant correlation are included.

Beetle assemblages in bolts differed among management strategies (p<0.01) and that result was consistent for the same beetle assemblage extracted from the window traps. Assemblages from clear-cuts differed from all other stand types (Table 2, Fig 4). Thinned stands supported significantly different assemblages from reference stands. There were no significant differences in assemblages between thinning, selective felling or old growth stands. However the difference between thinning and selective felling was marginally non-significant for window traps (p = 0.09). We did not find any differences between selective felling, old growth and reference stands. Stand size and altitude did not influence the species assemblage from bolts; but both influenced captures from the window traps (Table 1).

**Table 2:** ManyGLM analyses. Differences in beetle assemblage between stands with different management strategies for data from bolts, window traps and early (decay class preference below 3; dead wood still hard) and late successional species (decay class preference 3 and above; wood has started to soften), respectively. Overall results from manyGLM are presented at the top of the table, followed by the effect of management strategy from the pairwise comparisons.

	Bolts			Window trap comparison			Early species			Late species		
	Deviance	p	DF	Deviance	p	DF	Deviance	p	DF	Deviance	p	DF
Management strategy	178	**<0.01**	34	310	**<0.01**	33	575	**<0.01**	33	951	**<0.01**	33
Stand size	25	0.75	33	67	**<0.01**	32	127	**0.01**	32	133	0.2	32
Altitude	44	*0*.*09*	32	53	**0.03**	31	134	**0.01**	31	210	**<0.01**	31
Pairwise comparisons	Deviance	p	DF	Deviance	p	DF	Deviance	p	DF	Deviance	p	DF
CC vs T	65.6	**<0.01**	11	136.9	**<0.01**	10	213.4	**0.02**	10	357.8	**<0.01**	10
CC vs SF	54.6	**<0.01**	15	147.3	**<0.01**	14	208.2	**<0.01**	14	387.4	**<0.01**	14
CC vs R	55.9	**<0.01**	13	142.5	**<0.01**	12	213.8	**<0.01**	12	340.1	**<0.01**	12
CC vs OG	70.5	**<0.01**	14	118.8	**<0.01**	13	220.8	**<0.01**	13	389.3	**<0.01**	13
T vs SF	41.0	0.14	10	66.0	*0*.*09*	10	98.2	0.30	10	193.7	*0*.*10*	10
T vs R	34.3	**0.05**	9	56.5	**0.03**	9	82.3	**0.05**	9	130.0	**0.05**	9
T vs OG	35.4	0.29	9	69.9	0.14	9	114.6	0.20	9	183.5	*0*.*10*	9
SF vs R	19.4	0.41	12	32.8	0.19	12	70.2	0.13	12	109.7	0.11	12
SF vs OG	29.4	0.13	13	12.0	0.9	13	61.7	0.32	13	117.0	0.12	13
R vs OG	31.2	*0*.*10*	11	33.2	0.17	11	89.0	**0.04**	11	85.1	0.22	11

SF = selective felling. CC = clearcut T = thinning. R = reference, OG = Old growth. Bold numbers highlight significant p-values. Italics highlight marginally significant p-values.

### Comparison of the species patterns between bolt and window trap

Although we found similar statistical differences between forest types for both bolts and window traps, different species explained the differences in assemblage composition. The overall comparisons showed the differences, species assemblage on clear-cut differed from all other treatments and assemblages in thinning differed from the assemblage in reference stand. However, the univariate GLMs (S1 Table), showed no shared species with significant effects from management strategy between trapping methods. Five species from bolts differed significantly with different management strategies. The fungivore species *Leptusa pulchella*, was more abundant in old growth forest than in reference stands, and more abundant in reference stands than clearcuts. All other species showing differences were cambium consumers with a preference for early decay stages. *Orthotomicus suturalis* was highly abundant on the clearcuts but missing elsewhere. *Hylastes cunicularius* was less abundant in clearcuts and thinned stands compared with reference stands. *Pityogenes chalcographus* was most abundant on clearcuts, with decreasing abundances in thinning, selective felling, old growth and reference stands. *Polygraphus subopacus* was only captured in bolts in thinned stands.

**Fig 5:**
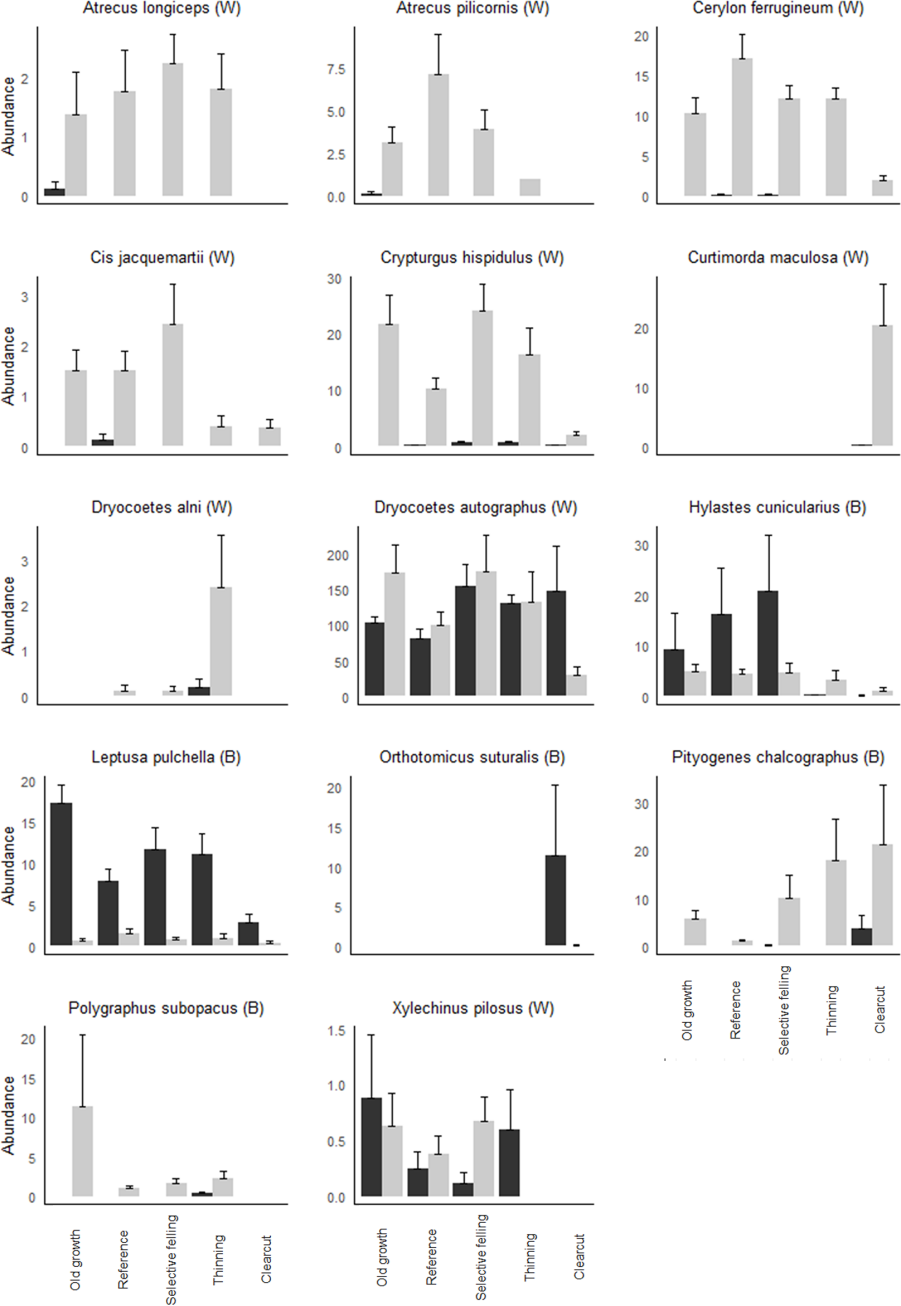
Species with significant treatment effects. Mean ± abundance by management strategy among management strategies for species captured using bolts (black bars, marked with a B if significant) and window traps (grey bars, marked with W if significant). Only species that differed significantly are shown, but results from both trapping methods are always shown. Note the variable scale on the y-axis.

Of the nine species affected significantly by management strategy in the window trap data, four were predators, three fungivores and two cambivores. Two of the fungivores (*Cis jacquemartii* and *Curtimorda maculosa*) and one predator (*Atrecus longiceps*) preferred late decay stages. The highly abundant cambivore species *Dryocoetes autographus* was less abundant in reference stands. The predators *Atrecus pilicornis* and *Atrecus longiceps* were absent from clearcuts. The cambivore *Xylechinus pilosus* occurred only in selective-fellings, reference and old growth forests and the fungivore *Cis jacquemartii* had its main distribution in selective-fellings, reference and old growth forests. The fungivore species *Curtimorda maculosa* occurred almost exclusively in clearcuts (Fig 5). *Pityogenes chalcographus* and *Polygraphus subopacus* showed marginally non-significant responses to management strategy.

Some general trends in species distributions were consistent for both sampling methods: species with high abundance on clear-cuts tended to occur in low abundances in the other stand types, while species with high abundances in reference and old growth stands tended to occur in low abundances, or were absent from the clear-cuts. Some species, common in old growth and reference stands, were less abundant in thinning (Fig 5).

### Response of early and late deadwood-associated beetles to different forest management strategies

Assemblages of early successional species showed a similar response to management strategy as the assemblages from the bolts (Table 1). Clear-cuts differed from all other stand types. Thinning and reference stands had significantly different species composition. We saw minor differences in how the early and the late assemblages responded to management strategy. Assemblages of early saproxylic beetles differed between reference and old growth stands. Late successional species showed marginally non-significant differences between thinning and selective felling and thinning and old growth stands (p = 0.10).

### Influence of stand variables and deadwood on beetle communities

As an effect of the selection process, altitude, stand size and proportion of spruce and birch did not differ among sites with management strategies (Table 1, Fig 1). Clear-cuts had the lowest average basal area, since most of the old trees had been removed, followed by selective felling and thinning. Reference and old growth stands had the highest basal area. Deadwood volumes also differed among stand types, with the highest volumes in old-growth forests. Thinned and clear-cut stands had the lowest volumes (Table 1).

Basal area had the strongest relationship with assemblage composition for both early and late successional species (r^2^ = 0.5, p <0.01 and r^2^ = 0.7, p <0.01, respectively), followed by the proportion of birch and standing deadwood of early decay-classes. The composition of beetle assemblages from experimental bolts was correlated with basal area and early decay classes of deadwood, both standing and lying (logs) (Table 3, Fig 4).

**Table 3:** Fitted stand variables to the NMDS matrix. The tables list the stand variables and the fit (r^2^) to the NMDS plot if the variable had a significant influence on the species assemblage. This is listed for all four data sets; from bolts, window traps, early (decay class preference below 3; dead wood still hard) and late successional species (decay class preference 3 and above; wood has started to soften). The top table includes all stand types (clear-cut, thinning, selective-felling, reference and old-growth). Clear-cuts are excluded from table B.

A) All stands								
	Bolts		Trap comparisson		Early species		Late species	
	r^2^	p	r^2^	p	r^2^	p	r^2^	p
Stand size (ha)	0.07	0.24	0.13	*0*.*08*	0.15	*0*.*06*	0.08	0.21
Altitude (msl)	0.05	0.41	0.07	0.26	0.12	*0*.*09*	0.06	0.30
Spruce (%)	0.09	0.20	0.02	0.76	0.03	0.60	0.02	0.71
Birch (%)	0.03	0.56	0.14	*0*.*07*	0.21	**0.02**	0.17	**0.04**
Basal area (m^2^)	0.52	**<0.01**	0.50	**<0.01**	0.50	**<0.01**	0.74	**<0.01**
X (latitude)	0.02	0.71	0.05	0.40	0.07	0.29	<0.01	1.00
Y (longitude)	0.02	0.62	0.10	0.14	0.07	0.29	<0.01	0.93
Early standing	0.20	**0.03**	0.14	*0*.*06*	0.21	**0.02**	0.15	**0.05**
Early logs	0.19	**0.03**	0.11	0.13	0.17	*0*.*06*	0.03	0.58
Late standing	0.12	*0*.*08*	0.11	0.14	0.12	0.12	0.11	0.12
Late logs	0.06	0.30	0.01	0.78	0.05	0.33	0.01	0.88
B) Without clearcuts								
	Bolts		Trap comparisson		Early species		Late species	
	r^2^	p	r^2^	p	r^2^	p	r^2^	p
Stand size (ha)	0.01	0.96	0.16	*0*.*10*	0.18	**0.05**	0.02	0.77
Altitude (msl)	0.08	0.32	0.26	**0.01**	0.25	**0.03**	0.16	*0*.*07*
Spruce (%)	0.01	0.96	0.28	**0.01**	0.18	*0*.*07*	0.13	0.14
Birch (%)	0.11	0.20	0.29	**0.01**	0.30	**<0.01**	0.03	0.65
Basal area (m^2^)	0.11	0.21	0.01	0.82	0.03	0.66	0.11	0.21
X (latitude)	0.15	0.12	0.11	0.19	0.13	0.14	0.05	0.52
Y (longitude)	0.09	0.30	0.03	0.68	0.01	0.87	0.03	0.65
Early standing	0.12	0.16	0.08	0.31	0.13	0.13	0.12	0.17
Early logs	0.24	**0.02**	0.14	0.13	0.18	*0*.*07*	0.12	0.19
Late standing	0.08	0.30	0.07	0.37	0.06	0.37	0.02	0.71
Late logs	0.14	0.11	0.10	0.26	0.12	0.17	0.08	0.33

After removing the clear-cuts, the influence of basal area disappeared for all groups. Stand size, altitude and proportion of birch influenced the early successional species, while the proportion of spruce and early decay-stage logs had a marginally non-significant effect. The composition of beetle assemblages in bolts was still associated with the volume of logs in early decay stages. None of the stand variables influenced late successional beetle assemblage (Table 3).

## Discussion

### Forest management strategy affects saproxylic assemblages

We asked how management strategy influences biodiversity, focusing on the saproxylic beetle assemblage, a group that is highly sensitive to the transformation of forest by management [54]. Management strategy altered the species assemblages. Our experimental design allowed us to evaluate both the difference between even and uneven-aged forests and the effect of time since clear-cutting in the even-aged system. Consistent with previous studies, we reported that the species composition on recent clear-cuts differed from all older forest types, [12, 25] and that results are consistent regardless of sampling method and deadwood decay-stage preference. The most likely explanation for this is that the removal of tree cover alters microclimate and thereby modifies sun-exposure, moisture levels and the decay rate of the deadwood, all of which are known to affect the saproxylic community [35, 52, 55]. In fact, even small-scale variation in light and temperature within an uneven-aged managed stand can have a strong impact on beetle assemblages with gaps favouring light demanding species [56].

Most species differed between clear-cuts and all other stand types: species favoured by clear-cuts were normally rare or absent in the elsewhere and vice versa. Some species, such as *Hylastes cunicularius* and *Pissodes gyllenhalii*, which were highly abundant in the uneven-aged stands, had lower abundance in thinning. However, we saw recovery over time in the even-aged stands. As the canopy cover recovered after clear-cutting, the beetle assemblages followed. Approximately 50 years after clear-cutting, i.e., in the thinned stands, beetle assemblages resembled the uneven-aged stands more closely. Nonetheless, significant differences between thinning and reference stands indicate that beetle composition has yet to recover fully to ‘pre-harvest’ conditions.

In contrast to clear-cutting, selective felling maintained a beetle assemblage similar to old growth and reference stands. Thus, uneven-aged silviculture has the potential to maintain the assemblage of the unharvest forest despite ongoing wood extraction. However, the early successional species and the assemblage captured in the bolts did not differ from the thinned stands. The strong recovery of the species pool in thinned stands suggests a resilient species pool [57]. However, we cannot exclude the possibility that this species pool has been already been filtered by management at the landscape scale, such that assemblages are dominated by disturbance favoured species [58]. Intensive even-aged silviculture has been occurring in our study landscape for at least 60–70 years, which might already have affected the species pool on a landscape level by filtering out or lowering the abundances of the species most sensitive to forest management. The old growth forests, set aside due to their high nature conservation values, provided the highest deadwood volumes and greatest proportion of large trees. Nevertheless, we could not detect differences in beetle composition between those stands, selective-fellings and thinning. A relatively large proportion of the species were present in several stand types. Stands with tree cover shared about 40% of their species, indicating a species pool with rather general habitat requirements. Still, the differences in assemblage composition in between clear-cut and thinned stands have implications for landscape-scale biodiversity. In a study in temperate forest [59] reported that, on the landscape scale, even-aged stands had higher gamma diversity than uneven-aged stands, a consequence of even-aged stands hosting different assemblages during different age stages. The situation is probably similar in boreal forest as uneven-age forest management only mimics one type of natural disturbance in the boreal forest, the small-scale stand disturbance. Thus, other types and scales of disturbances need to be incorporated into landscape-scale management [22].

Early successional beetles living off the rapidly consumed phloem of recently killed trees are generally considered capable of long and fast movement by flight and therefore least likely to be threatened by forestry [60, 61]. The quality of the surrounding habitat might therefore be of lesser importance than the quality of the cambium for these species [31, 60, 62] Seventy-five percent of species and ninety-five percent of the individuals collected from the bolts were early successional species. Woody debris and low stumps from harvest operations create (generic) deadwood in managed stands, which might explain why there was no detectable difference between old growth, selective-felling and thinning. However, since we were unable to link the amount of deadwood to species composition, we cannot confirm this hypothesis.

Late successional species are thought to be more dispersal limited, often having more specific habitat demands [8, 60, 61] and we predicted a stronger response to management strategy for late successional species. However, we found no support for this prediction: differences among stand types were similar between early and late successional species. Compared with the rapidly decomposing cambium, the later decay stages provide habitat for a longer time [31, 63] and remnants of old deadwood might act as a buffer, even in the stands with low levels of old wood. The assemblages of late successional saproxylic beetles in thinned stands were (marginally non-significantly) different from selective felling and old growth forest. Low levels of replication for the thinned treatment may have decreased statistical power and a larger number of stands could alter the results. However, the trend of species composition in thinning differing from selective-felling and old-growth forests as well as from reference stands could indicate that even-aged management had an stronger influence on the assemblage of on late successional species more, while influencing early successional species less.

We collected few red-listed species for any stand type, making statistical tests of impacts on these taxa impossible. Sampling rare species requires large samples, at least 200 species or 2000 individuals has been suggested [64]. In this study, over 20 000 individuals and 220 species were collected, but red-listed species made up only 3.8% of the species and 0.1% of individuals. Thus, the sample size for red-listed beetles was so small that statistical analyses for that group would likely have had insufficient power to detect differences among treatments. The low numbers of red-listed beetles highlights the question of whether the landscape after decades of intensive forest management no longer supports rare, specialised species [58, 65] but instead an impoverished species pool and reduced variation in assemblage composition between stand types. Targeted sampling to evaluate the current pool of red-listed species across stand types could allow us to test this proposition.

### Consistency between sampling methods

Patterns in beetle communities were mostly consistent, regardless of sampling method. We saw the same significant difference between the different management strategies for both bolts and window traps. Thus, our findings support the assumption that a general trapping method (such as window trapping) can be consistent with a more targeted trapping method (emergence trapping, bark sieving) and that both methods reflects the beetle assemblage of the surrounding stand [37, 38].

However, even though both trapping methods showed a similar treatment response, the difference explained by different species depended on trapping method. None of the species that explained the treatment response were shared between both sampling methods. Preferred breeding substrate is likely a major determinant of species abundances from different trap types, and species captured in bolts were only those that could develop in spruce deadwood of early decay stage. The assemblage captured in window traps was not constrained by substrate preference, but species might occur in different abundances depending on substrate availability at a larger scale. Birkemoe and Sverdrup-Thygeson [66] detected differences in the proportion of feeding guilds between direct and general trapping methods. In particular, more fungivores and fewer predators were trapped in window traps than in emergence traps. In our study, both bolts and early successional assemblages were dominated by cambivores which might explain the similarities between trap types. We chose to expose that bolts over two seasons and by doing so we might lose some of the earliest colonisers but gain species colonising later. One example of early colonisers is *Trypodendron lineatum* Earlier studies of boreal spruce forest in Sweden showed a strong drop in abundance of from year one to year two, [67, 68] and *T*. *lineatum* was missing from our study.

### Differences between early and late deadwood associated beetles

We found minor differences in the responses of early and late successional beetles to forests with different management strategies, as discussed above. The main difference between the early and late successional species was, however, the shift in the proportion of feeding guilds represented. Diversity of deadwood decay type and stages is a key driver of saproxylic beetle diversity, with different dead wood types and decay stages providing habitat and food resources for different assemblages [54]. Cambium consumers dominated the early successional community, while fungivores dominated the late decay community. This is consistent with observed successional patterns in saproxylic assemblages [8, 61]. It implies that, to maintain diversity of saproxylic beetles, there is a need for both early and late decay deadwood to exist concurrently. Since it can take considerable time for late decay stages of deadwood to form, it is crucial to consider deadwood recruitment during all stages of forest management [31]. Selective felling might maintain suitable habitat for saproxylic beetles by providing a more even deadwood input since mature trees are present continuously, much like in a late successional forest [9, 69]. However, to achieve this goal, it is crucial that sufficient numbers of mature and senescent trees are left in a stand.

### Deadwood availability influenced beetle communities

Basal area was strongly correlated with the species composition, but only when clear-cutting was included. This suggests that the low basal area/tree cover was the most important driver of the differences between species assemblages on clear-cuts and other stand types [52, 55]. Deadwood volume was correlated with assemblage composition in bolts and the early successional species. The volume of early decay-stage deadwood was most important, indicating that quality of deadwood needs to be considered alongside deadwood volume [8, 39, 52]. Surprisingly, we saw no correlation between the volume of late decay classes of wood and species associated with late decay stages of deadwood. The composition of living trees was also an important variable for beetle assemblages, with the volume of birch an important correlate of the early successional species community. Species associated with the early decay stages tend to be more specific to hosts [8], which might explain this correlation. Both altitude and latitude influenced the assemblage, indicating local differences in the species pool. However, this trend was mainly visible for species associated with the early decay stages. The composition of late successional species was largely unaffected by the stand variables. Overall, the strong relationships between environmental variables and beetle species assemblages suggest that the present-day characteristics of stands are important in regulating assemblage composition. This indicates an avenue through which foresters might manipulate production forests to favour particular biodiversity outcomes.

### Conclusions

This study shows that forest management strategy influences the saproxylic beetle assemblage. Even-aged silviculture alters the species assemblage dramatically in the short-term, but assemblages converge on those of uneven-aged silviculture as forests regrow. Fifty years after clearcutting, the species assemblages still differ from reference stands and the potential of those stands to act as a biodiversity source are likely limited. Beetle community composition in an even-aged managed landscape will likely be driven by the proportion of area clear-cut per year and the beetle recolonization rates as the forest regrows. Uneven-aged silviculture maintains the species assemblage of the mature or old forest and may be a better management option than even aged silviculture if biodiversity conservation for old growth -associated species is considered. However, the long-term effect of uneven-aged silviculture needs to be evaluated in stands subjected to several cycles of active uneven-aged silviculture. Repeated harvesting might lower the deadwood supply and therefore affect the beetles. However, uneven-age forest management only mimics one type of natural disturbance in the boreal forest: small-scale stand disturbances. Thus, for landscape scale management, large-scale disturbances, e.g. fire, also need to be considered.

Saproxylic species are dependent on the deadwood resource and the longer-term effects of even-aged silviculture resulted in supressed volumes of deadwood, especially of the late decay stages. Surprisingly, early and late successional species responded similarly to forest management strategy. One reason for this could be that the landscape may not support that many rare, demanding species after so many years of intense management. We suggest that future studies address this important gap in knowledge by using targeted sampling for red-listed species.

## Supporting information

S1 Table Species list over bark sample species, matching abundances from window traps and the ManyGLM output. (PDF)

S2 Table Species list from window traps and the abundance per treatment. (PDF)
