# Supplementary material for: Forest management strategy affects saproxylic beetle assemblages: A comparison of even and uneven-aged silviculture using direct and indirect sampling
Source: PLoS One. 2018 Apr 10;13(4):e0194905. doi: 10.1371/journal.pone.0194905 (PMC5892898; doi:10.1371/journal.pone.0194905)
Supplement: S1 Table — (PDF) [file pone.0194905.s001.pdf]

S1 Table

S1 Table. Species list over bark sample species and the matching abundances from window traps. Scientific names follow the Swedish taxonomic database (Dyntaxa version 1.1.6102.24188, 2015) Feeding guild were determined from previous research (Koch 1989, Palm 1959). F= fungivore, C= cambivore, P= predator.

			**Abundance bark sample**					**ManyGLM output**		**Abundance window traps**					**ManyGLM output**	
Species	Family	Feeding guild	CC	T	SF	R	OG	t-stat	p value	CC	T	SF	R	OG	t-stat	p value
Anomognathus cuspidatus	Staphylinidae	P	1	1	2	2		3.76	0.544	8	5	6	3	3	2.25	0.815
Atrecus longiceps	Staphylinidae	P				1		3.17	0.32		13	20	14	11	17.39	0.006
Atrecus pilicornis	Staphylinidae	P				1		3.17	0.324		10	35	57	25	35.28	0.001
Cerylon ferrugineum	Cerylonidae	F			1		1	3.33	0.502	16	70	109	137	82	35.59	0.001
Cis jacquemartii	Ciidae	F			1			3.17	0.384	3	3	22	12	12	12.71	0.021
Crypturgus hispidulus	Curculionidae	C	1	3	1		5	7.36	0.188	16	93	217	81	173	30.90	0.001
Curtimorda maculosa	Mordellidae	F	1					2.93	0.633	167		1			35.52	0.001
Dadobia immersa	Staphylinidae	F	2					3.08	0.613				1	2	3.85	0.356
Dinaraea aequata	Staphylinidae	F	1				2	3.45	0.491		3				4.37	0.127
Dryocoetes alni	Curculionidae	C	1					2.93	0.584		12	1	1		14.57	0.006
Dryocoetes autographus	Curculionidae	C	1327	884	648	825	1392	2.71	0.593	234	684	1573	793	1392	11.75	0.02
Homalota plana	Staphylinidae	F	1	1	2	1	3	1.40	0.904	1					3.11	0.507
Hylastes brunneus	Curculionidae	C	75	210	72	159	86	9.46	0.177	35	83	74	34	57	6.23	0.34
Hylastes cunicularius	Curculionidae	C	1	5	130	74	187	14.86	0.028	9	17	41	35	39	6.41	0.197
Hylobius abietis	Curculionidae	C	1					2.93	0.564	2					6.23	0.102
Hylobius excavatus	Curculionidae	C			1			3.17	0.396				1		3.12	0.447
Hylobius pinastri	Curculionidae	C	3					6.38	0.102	2		1			5.29	0.215
Hylurgops palliatus	Curculionidae	C		1	4	16	3	9.39	0.126				1		3.12	0.415
Leptusa pulchella	Staphylinidae	F	26	62	63	138	105	23.53	0.001	3	7	7	12	5	4.88	0.394
Orthotomicus suturalis	Curculionidae	C	102					16.79	0.003	1					3.12	0.512
Pissodes gyllenhalii	Curculionidae	C	3	15	51	57	52	7.79	0.142	1					3.12	0.512
Pityogenes chalcographus	Curculionidae	C	33				2	9.23	0.055	169	91	89	8	46	13.04	0.072
Polygraphus subopacus	Curculionidae	C		2				8.22	0.017		11	14	8	90	14.28	0.067
Pteryx suturalis	Ptiliidae	F		2		1		4.50	0.223	121	120	350	256	248	4.21	0.252
Quedius plagiatus	Staphylinidae	P				1		3.17	0.324	3	18	29	26	20	8.88	0.065
Rhagium inquisitor	Cerambycidae	C	1					2.93	0.598	1		2		1	2.89	0.832
Rhyncolus ater	Curculionidae	C			2		1	3.77	0.401		2	1		1	5.79	0.211
Xylechinus pilosus	Curculionidae	C		3	2	7	1	8.67	0.114			6	3	5	12.42	0.019
