# Supplementary material for: Forest management strategy affects saproxylic beetle assemblages: A comparison of even and uneven-aged silviculture using direct and indirect sampling
Source: PLoS One. 2018 Apr 10;13(4):e0194905. doi: 10.1371/journal.pone.0194905 (PMC5892898; doi:10.1371/journal.pone.0194905)
Supplement: S2 Table — (PDF) [file pone.0194905.s002.pdf]

S2 Table

S2 Table. Species list from window traps. Scientific names follow the Swedish taxonomic database (Dyntaxa version 1.1.6102.24188, 2015)

Feeding guild were determined from previous research (Koch 1989, Palm 1959). F= fungivore, C= cambivore, P= predator, W=wood-borer.

Decay niches are adapted from Thorn et. al 2014

Species	Family	Decay niche	Feeding guild	Clearcut	Thinning	Selective	Reference	Old-growth
Abdera affinis	Melandryidae	3,3	F	2				
Abdera flexuosa	Melandryidae	3,3	F	1		2		
Alosterna tabacicolor	Cerambycidae	3,6	W		1	4		
Ampedus balteatus	Elateridae	3,4	P	26				
Ampedus nigrinus	Elateridae	3,7	P	129	8	1	9	3
Ampedus pomonae	Elateridae	3,5	P	2				
Ampedus tristis	Elateridae	3,4	P	309		3	1	
Anaspis bohemica	Scraptiidae	4,0	P	13	2			1
Anaspis rufilabris	Scraptiidae	4,0	P	1	2	3	1	5
Anaspis thoracica	Scraptiidae	4,0	F	1				
Anastrangalia reyi	Cerambycidae	3,0	W	3				
Anisotoma axillaris	Leiodidae	4,0	F	72		4	1	1
Anisotoma castanea	Leiodidae	4,0	F	5		1	2	1
Anisotoma glabra	Leiodidae	4,0	F	53	13	14	5	8
Anisotoma humeralis	Leiodidae	4,0	F	3	3	8	4	10
Anisotoma orbicularis	Leiodidae	4,0	F	13	1	4	3	1
Anomognathus cuspidatus	Staphylinidae	2,8	P	8	5	6	3	3
Anthaxia quadripunctata	Buprestidae	1,8	C	13				
Aplocnemus tarsalis	Melyridae	2,5	P	2				
Arhopalus rusticus	Cerambycidae	2,5	C	1				
Atomaria bella	Cryptophagidae	3,0	F	14	18	28	17	61
Atomaria vespertina	Cryptophagidae		F		4		8	28
Atrecus affinis	Staphylinidae	4,0	P		2			
Atrecus longiceps	Staphylinidae	3,5	P		13	20	14	11
Atrecus pilicornis	Staphylinidae	2,0	P		10	35	57	25
Bibloporus bicolor	Staphylinidae	3,4	P	37	57	97	74	48
Bolitochara mulsanti	Staphylinidae	3,5	F		1			3
Bolitophagus reticulatus	Tenebrionidae	3,3	F	4				
Cacotemnus rufipes	Anobiidae		W					1
Callidium coriaceum	Cerambycidae	2,0	C	2				
Calopus serraticornis	Oedemeridae	3,4	W	1	1		1	
Cerylon deplanatum	Cerylonidae	2,0	F		4	2		
Cerylon ferrugineum	Cerylonidae	2,4	F	16	70	109	137	82
Cerylon histeroides	Cerylonidae	3,7	F	78	87	130	110	144
Chrysanthia geniculata	Oedemeridae		W	5				
Cis bidentatus	Ciidae	3,4	F		1	6	3	13
Cis boleti	Ciidae	3,4	F	212	15	26	12	17
Cis castaneus	Ciidae	3,4	F		1	3	1	4
Cis comptus	Ciidae	3,3	F	29	6	11	4	3
Cis dentatus	Ciidae	3,3	F	2	4	4	4	17
Cis festivus	Ciidae		F		1	2		
Cis glabratus	Ciidae	3,3	F		2	3		
Cis jacquemartii	Ciidae	3,3	F	3	3	22	12	12
Cis lineatocribratus	Ciidae	3,4	F	1	1	1		
Cis micans	Ciidae	3,3	F		7	5	1	3
Cis punctulatus	Ciidae	3,3	F	7	3	10		6
Cis quadridens	Ciidae	3,3	F		1			1
Cis submicans	Ciidae		F		1			
Cis vestitus	Ciidae		F	4	2	1	1	5
Corticaria lapponica	Corticariidae	3,0	F		2	3		
Corticeus linearis	Tenebrionidae	1,0	P	3				

Cryphalus saltuarius	Curculionidae	2,0	C		1	1		
Cryptolestes abietis	Laemophloeidae	2,0	P		1	2	1	2
Cryptophagus quadrihamatus	Cryptophagidae		F					1
Cryptophagus quercinus	Cryptophagidae	4,1	F		1			
Crypturgus cinereus	Curculionidae	2,0	C	10	11	62	15	504
Crypturgus hispidulus	Curculionidae	2,0	C	16	93	217	81	173
Curtimorda maculosa	Mordellidae	3,5	F	167		1		
Dacne bipustulata	Erotylidae	3,2	F	63			2	1
Dadobia immersa	Staphylinidae	2,5	F				1	2
Danosoma conspersum	Elateridae		P	2				
Danosoma fasciatum	Elateridae		P	30				
Dasytes niger	Melyridae	3,2	P	60				
Dasytes obscurus	Melyridae	3,2	P	2				
Dasytes plumbeus	Melyridae	3,2	P					1
Dendroctonus micans	Curculionidae	2,0	C		1	1	1	
Dendrophagus crenatus	Silvanidae	2,5	F	4	14	15	23	26
Denticollis borealis (NT)	Elateridae	3,7	P	2				
Denticollis linearis	Elateridae	3,4	P	6	4	8	1	1
Diacanthous undulatus	Elateridae	3,4	P	1	1		1	1
Dictyoptera aurora	Lycidae		P	8	10	23	17	24
Dinaraea aequata	Staphylinidae	3,5	F		3			
Dinaraea arcana	Staphylinidae	3,3	F		2	4	3	
Dinaraea linearis	Staphylinidae	3,5	F					1
Dolichocis laricinus	Ciidae		F		1			2
Dorcatoma dresdensis	Anobiidae	3,3	F	9	1	1	2	1
Dorcatoma robusta	Anobiidae	3,3	F	3	1	1		
Dropephylla clavigera	Staphylinidae		P	1				
Dropephylla linearis	Staphylinidae		F,P	3	3	5	2	7
Dryocoetes alni	Curculionidae	2,0	C		12	1	1	
Dryocoetes autographus	Curculionidae	2,0	C	234	684	1573	793	1392
Eblisia minor (NT)	Histeridae	3,5	P	5				
Elateroides dermestoides	Lymexylidae		F	8	3	29	16	8
Endomychus coccineus	Endomychidae	3,4	F			4	1	2
Enicmus apicalis	Corticariidae		F			1	3	1
Enicmus planipennis (NT)	Corticariidae	3,5	F					1
Enicmus rugosus	Corticariidae	3,9	F	56	47	57	28	36
Enicmus testaceus	Corticariidae	3,0	F		3	1		
Ennearthron cornutum	Ciidae	3,4	F	3		9	1	
Episernus angulicollis	Anobiidae	2,5	W	1		2	1	
Eudectus giraudi	Staphylinidae		F		23	13	17	5
Euplectus punctatus	Staphylinidae	3,0	P	46	31	48	41	47
Glischrochilus quadripunctatus	Nitidulidae	2,0	F,P	10	5	10	2	1
Globicornis emarginata	Dermestidae		D	6				
Gnathacmaeops pratensis	Cerambycidae		C	1				
Gyrophaena boleti	Staphylinidae	3,3	F	5	3	3	4	4
Gyrophaena minima	Staphylinidae	3,7	F			1		
Hadreule elongatula	Ciidae		F	7		1		1
Hadrobregmus pertinax	Anobiidae		C	36	1	1	1	2
Hallomenus axillaris	Melandryidae	3,0	F	1	2	5	1	3
Hallomenus binotatus	Melandryidae	3,4	F	3	15	20	11	23
Homalota plana	Staphylinidae	1,5	F	1				
Hylastes brunneus	Curculionidae	2,0	C	35	83	74	34	57
Hylastes cunicularius	Curculionidae	2,0	C	9	17	41	35	39
Hylobius abietis	Curculionidae	2,0	C	2				
Hylobius excavatus	Curculionidae		C				1	
Hylobius pinastri	Curculionidae	2,0	C	2		1		
Hylurgops palliatus	Curculionidae	2,0	C				1	
Ips typographus	Curculionidae	1,8	C	3	10	30	2	7

Judolia sexmaculata	Cerambycidae	3,4	C	26	3	4		
Latridius hirtus	Corticariidae	4,0	F	5	3	5	3	7
Leptophloeus alternans	Laemophloeidae	2,0	P			1		
Leptura quadrifasciata	Cerambycidae	3,3	W	7				
Lepturobosca virens	Cerambycidae	2,0	W	1				
Leptusa pulchella	Staphylinidae	3,8	F	3	7	7	12	5
Litargus connexus	Mycetophagidae	2,8	F	3				
Lordithon speciosus	Staphylinidae	3,7	P		7	7	11	9
Lordithon trimaculatus	Staphylinidae	3,7	P		1		1	
Magdalis frontalis	Curculionidae	2,0	W	1				
Malthinus biguttatus	Cantharidae	3,2	P		1	1		
Malthodes fuscus	Cantharidae	3,2	P	1	1			2
Melanotus castanipes	Elateridae	3,7	p	48	28	33	9	17
Melanotus villosus	Elateridae			17	14	10	1	6
Microscydmus minimus	Scydmaenidae	4,5	P	5		8	9	1
Molorchus minor	Cerambycidae	2,0	C	3		4		
Mordella aculeata	Mordellidae	3,0	F	2				
Mycetochara maura	Tenebrionidae		F		1		1	
Mycetochara obscura	Tenebrionidae	4,1	?	1	1			
Mycetophagus fulvicollis (NT)	Mycetophagidae	3,8	F	2	1			
Mycetophagus multipunctatus	Mycetophagidae	3,5	F	2	1	1		
Mycetophagus populi	Mycetophagidae	3,6	F					1
Octotemnus glabriculus	Ciidae	3,4	F	1	1	4	1	1
Olisthaerus megacephalus	Staphylinidae		P			1		
Orchesia fasciata (NT)	Melandryidae	3,4	F			4		3
Orchesia micans	Melandryidae	3,3	F		1	2		3
Orthocis alni	Ciidae	2,8	F	6	2	9	6	4
Orthotomicus laricis	Curculionidae	2,0	C	1		1		1
Orthotomicus suturalis	Curculionidae	2,0	C	1				
Oxymirus cursor	Cerambycidae	3,6	W	1			1	1
Pachyta lamed	Cerambycidae	2,5	C	1				
Pediacus fuscus	Cucujidae	2,0	F	1				1
Peltis ferruginea	Trogossitidae		F	3	1	4	1	3
Phloeonomus punctipennis	Staphylinidae	2,5	P		2	6	1	2
Phloeopora testacea	Staphylinidae	2,3	P	1				
Phloeostiba lapponica	Staphylinidae		P	9		4	1	
Phloeotribus spinulosus	Curculionidae		C	3	7	3	13	13
Phyllodrepa melanocephala	Staphylinidae	4,0	F,P	2				2
Pissodes gyllenhalii	Curculionidae	2,0	C	1				
Pissodes harcyniae	Curculionidae	2,0	C			1		3
Pissodes pini	Curculionidae	2,0	C	1				
Pityogenes bidentatus	Curculionidae	2,0	C	4	2		1	
Pityogenes chalcographus	Curculionidae	1,8	C	169	91	89	8	46
Pityogenes quadridens	Curculionidae	2,0	C	2				
Pityophagus ferrugineus	Nitidulidae	2,0	P	2	8	1	1	3
Pityophthorus micrographus	Curculionidae	2,0	C		1			1
Placusa atrata	Staphylinidae	1,6	P	1				
Platycerus caprea	Lucanidae	3,4	W	2		1		
Platycis minutus	Lycidae	4,0	P			3	1	3
Platysoma angustatum	Histeridae	2,0	P	1				
Platysoma lineare (NT)	Histeridae	2,0	P	1				
Plegaderus vulneratus	Histeridae	2,0	P	3	1	3		3
Podistra schoenherri	Cantharidae		P	17	4	19	10	3
Pogonocherus fasciculatus	Cerambycidae	2,0	C	7		1	1	1
Polygraphus poligraphus	Curculionidae	2,0	C	4	59	91	64	116
Polygraphus punctifrons	Curculionidae	2,0	C					8
Polygraphus subopacus	Curculionidae	2,0	C		11	14	8	90
Pteryngium crenatum	Cryptophagidae	3,0	F		2	9	8	6

Pteryx suturalis	Ptiliidae	4,1	F	121	120	350	256	248
Ptilinus fuscus	Anobiidae	3,0	W	1				
Ptinella johnsoni	Ptiliidae	3,4	F			2		
Ptinella tenella	Ptiliidae	4,0	F	1		2		
Ptinus subpillosus	Anobiidae		C		1	5	3	7
Pyropterus nigroruber	Lycidae	4,0	P	4		4	2	
Pytho abieticola (VU)	Pythidea	3,0	P			1		
Quedius plagiatus	Staphylinidae	4,0	P	3	18	29	26	20
Rabocerus foveolatus	Salpingidae	2,0	P	5	2	1		1
Rhagium inquisitor	Cerambycidae	2,4	C	1		2		1
Rhagium mordax	Cerambycidae	2,3	C		2		1	1
Rhizophagus cribratus	Monotomidae	2,7	P	4		2		1
Rhizophagus depressus	Monotomidae	2,0	P					1
Rhizophagus fenestralis	Monotomidae		P	36	40	100	47	17
Rhizophagus ferrugineus	Monotomidae	2,0	P	3	3	7		17
Rhizophagus grandis (NT)	Monotomidae	2,0	P				1	
Rhizophagus nitidulus	Monotomidae	2,8	P	2	5	33	14	31
Rhopalodontus strandi	Ciidae		F	3		3		1
Rhyncolus ater	Curculionidae	3,0	W		2	1		1
Rhyncolus sculpturatus	Curculionidae	3,0	W	5		1		
Rusticoclytus rusticus	Cerambycidae		C	1				
Salpingus ruficollis	Salpingidae	2,3	P	2	11	8	11	17
Schizotus pectinicornis	Pyrochroidae	2,5	C		1			
Scolytus ratzeburgii	Curculionidae		C	14				
Serropalpus barbatus	Melandryidae	2,3	W					1
Silvanoprus fagi	Silvanidae	2,0	P	4	1	3	1	2
Soronia grisea	Nitidulidae		D	3	1			
Sphaeriestes stockmanni	Salpingidae	2,0	P	1				
Stenotrachelus aeneus	Stenotrachelidae	2,8	C	19		1		2
Stenurella melanura	Cerambycidae	3,4	W	8				
Stictoleptura maculicornis	Cerambycidae		D,W	1				
Sulcacis fronticornis	Ciidae	3,4	F	4	2			
Sulcacis nitidus	Ciidae		F	7			4	
Synchita humeralis	Colydiidae	3,3	F		1			
Tachyta nana	Carabidae	2,8	P	9				
Tetratoma ancora	Tetratomidae	3,0	F		2	1	1	1
Tetropium castaneum	Cerambycidae	2,0	C	6	2	4	1	8
Tetropium fuscum	Cerambycidae	2,0	C	1				2
Thanasimus femoralis	Cleridae	2,3	P			1	1	
Thanasimus formicarius	Cleridae	2,3	P	1				
Thiasophila wockii	Staphylinidae		P			1	1	1
Tomoxia bucephala	Mordellidae	3,0	W	1				
Trichius fasciatus	Scarabaeidae	3,5	W	2				
Triplax aenea	Erotylidae	3,4	F	9	12	11	15	6
Triplax russica	Erotylidae	3,4	F	15	1	3		
Triplax scutellaris	Erotylidae	3,4	F	1	10	22	41	18
Trypodendron domesticum	Curculionidae		F		1	3	1	
Trypodendron laeve	Curculionidae		F		16	7	12	3
Trypodendron lineatum	Curculionidae		F	5	75	126	115	154
Trypophloeus bispinulus	Curculionidae		C	1				
Wanachia triguttata	Melandryidae		F	8				
Xylechinus pilosus	Curculionidae	2,0	C			6	3	5
Xylita laevigata	Melandryidae	3,5	F	10	12	26	4	20
